# Pathogenicity, Mycotoxin Production, and Control of Potato Dry Rot Caused by *Fusarium* spp.: A Review

**DOI:** 10.3390/jof9080843

**Published:** 2023-08-12

**Authors:** Huali Xue, Qili Liu, Zhimin Yang

**Affiliations:** 1 College of Science, Gansu Agricultural University, Lanzhou 730070, China; 2 College of Food Science and Engineering, Gansu Agricultural University, Lanzhou 730070, China; lql744607@163.com; 3 Lanzhou Institutes for Food and Drug Control, Lanzhou 730070, China; yzmljh@163.com

**Keywords:** *Fusarium* spp., Fusarium dry rot of potato, pathogenicity, mycotoxin, control

## Abstract

Fusarium dry rot is one of the major potato diseases during storage after harvest, which not only results in quality degradation but also causes great economic losses. The disease can be elicited by some species of *Fusarium*, and the pathogenic fungi of *Fusarium* causing potato dry rot are considerably diverse in various countries and regions. The disease caused by *Fusarium* spp. is associated with mycotoxins accumulation, which has phytotoxic and mycotoxic effects on humans and animals. Chemical synthetic fungicide is considered the main control measure for the Fusarium dry rot of potato; nevertheless, it is unfortunate that persistent application inevitably results in the emergency of a resistant strain and environmental contamination. A comprehensive disease control strategy includes potato cultivar selection, appropriate cultural practices (crop rotation, cultivate pattern, fertilization, and irrigation), harvesting processes and postharvest treatments (harvesting, classification, packaging, wound healing), and storage conditions (environmental disinfection, temperature, humidity and gas composition) along with the application of fungicide pre-harvest or post-harvest. Recently, emerging studies have indicated that eco-friendly strategies include physical control, chemical methods (such as the application of generally-recognised-as-safe (GRAS) compounds or chemical (elicitors) and biological control have been introduced to combat the Fusarium dry rot of potato.

## 1. Introduction

Potato (*Solanum tuberosum* L.) ranks fourth as the most important food crop all over the world, only behind rice (*Oryza sativa* L.), wheat (*Triticum aestivum* L.), and maize (*Zeamays* L.). It is also a major non-cereal food crop, which plays an irreplaceable role in the global food supply. Potato is rich in plenty of macronutrients (such as carbohydrates and dietary fibre) and micronutrients (such as vitamins and minerals); furthermore, it is also an important source of antioxidants in people’s diet [1]. It is reported that, in 2020, the total world production of potatoes was 359 million tonness (http://www.fao.org/faostat/en/?#data/QC, accessed on 1 January 2021) and China ranked the first with the production of 17.98 million tons [2,3]. More than 85% of potatoes need to be kept for 3–6 months as a vegetable and industrial material, and the losses due to the disease during storage are very large [4]. Some fungi, bacteria, and viruses can result in post-harvest disease. Among them, the infection of potato tubers resulting from *Fusarium* spp. Can particularly cause severe dry rot during storage, which not only leads to quality deterioration but also reduces the marketable yield. Tuber losses range from 6.25% to 25% due to dry rot during storage annually, and up to 60% when potato tubers are injured [5]. In the Gansu province of China, it is estimated that tuber losses due to dry rot were around 88% of the total post-harvest losses [6]. Importantly, some *Fusarium* species associated with dry rot produce mycotoxins, which pose an adverse effect on humans and animals due to their mycotoxicoses.

Once the pathogenesis of *Fusarium* is known, appropriate and effective management measures can be carried out, and the losses will be effectively reduced. At present, some chemical synthetic fungicides (such as carbendazim, mancozeb, and thiabendazole (TBZ)) are employed to prevent and control the dry rot of potato. Nevertheless, chemical synthetic fungicides are not a long-term solution due to the ecological environment and drug resistance. Currently, some eco-friendly control measures such as the use of some organic acids and salts [7], inorganic salts [8,9], chitosan [10], plant essential oil [11,12] and biological antagonists [13,14] for the management of post-harvest disease in potato tubers are being explored and developed. The present review focuses on dry rot occurrence (including the causal pathogens of dry rot, the symptoms of dry rot, and its pathogenesis), mycotoxin production (non-trichothecenes and trichothecenes), and management strategies.

## 2. Dry Rot of Potato Tubers

### 2.1. The Causal Agent Causing Dry Rot

*Fusarium* is a notorious and large fungal genus within the Ascomycota phylum containing hundreds of species, which are primarily isolated from soil and plant survival [1]. It is well-known that *Fusarium* spp. have the ability to cause potato dry rot, which is a devastating form of post-harvest fungal decay, severely impacting potato tuber quality all over the world [1]. Fusarium dry rot causes a remarkable reduction in potato yield, as well as leading to enormous economic losses. Currently, there are 17 species, and 5 variants of *Fusarium* recognised globally as causal agents of potato dry rot [15]. Because of the differences in potato cultivars and climatic conditions, diverse *Fusarium* species were isolated and identified from the Fusarium dry rot of potato in various countries and regions. Among them, *F. sambucinum* was considered the most predominant pathogenic fungus leading to the Fusarium dry rot of potato in North America and some regions of Europe [16,17,18]; however, some reports suggested that *F. solani* var. *coeruleum* was regarded as the most prevalent pathogen causing potato dry rot under low-temperature storage in the United Kingdom, and occasionally, the pathogen of *F. coeruleum* also caused severe potato harvest disease in the United Kingdom [19,20,21]. *F. graminearum* was the most frequently *Fusarium* species caused potato dry rot in North Dakota [22]. *F. oxysporum* and *F. solani* were reported as the main pathogens to cause Fusarium dry rot in potato in South Africa [23,24]. *F. sulphureum* and *F. solani* were found to have a higher incidence and higher aggressiveness in Iran [25]. In Egypt, *F. sambucinum* was regarded as the most predominant pathogen, except for *F. oxysporum*, *F. incarnatum* and *F. verticillioide* [26] (Table 1).

There are different climatic conditions in China, and different species of *Fusarium* were isolated and characterised in different regions. Potato planting regions are divided into four planting regions in China, Northeast, North, Northwest, and South China. In the potato planting regions of Northwest China, *F. avenaceum*, *F. oxysporum*, *F. sporotrichiodes*, *F. solani*, *F. trichothecioides*, *F. solani* var. *coeruleum*, *F. sambucinum*, *F. semitectum*, *F. solani*, *F. sambucinum*, *F. culmorum*, *F. gibbosum*, *F. macroceras*, *F. solani* var. *coeruleum*, *F. acuminatum*, *F. equiseti* and *F. redolens* were identified in Heilongjiang Province and Inner Mongolia Autonomous Region [27]; in North China, *F. sambucinum* and *F. avenaceum*, *F. solani* var. *coeruleum*, *F. oxysporum*, and *F. acuminatum* were isolated in Shanxi Province [27]. In Northwest China, *F. sambucinum*, *F. avenaceum*, *F. oxysporum*, and *F. equiseti* were identified in Ningxia Hui Autonomous Region; *F. sambucinum*, *F. solani*, *F. sulphureum*, *F. avenaceum*, and *F. graminearum* were identified in Gansu Province [7,8,10,12]; *F. tricinctum*, *F. avenaceum*, *F. oxysporum*, *F. solani*, *F. acuminatum*, and *F. equiseti* were mainly identified in Qinghai Province [6]. In Zhejiang Province of South China, *F. solani*, *F. solani* var. *coeruleum*, *F. moniliforme var*. *intermedium*, *F. moniliforme var. zhejiangense*, and *F. redolens* were mainly identified [6] (Table 1).

The occurrence of potato dry rot is not only influenced by countries and regions; potato variety and chemical synthetic fungicide application, as well as seed tuber source also play significant roles. For instance, Xue’s research group [28] compared the pathogenicity of *F. sulphureum* for different potato cultivars, and found that the variety of Longshu No. 3 is susceptible, while the variety of Longshu No.6 is resistant for *F. sulphureum*.

### 2.2. Pathogen Infection and the Symptoms of Potato Dry Rot

**Figure 1:**
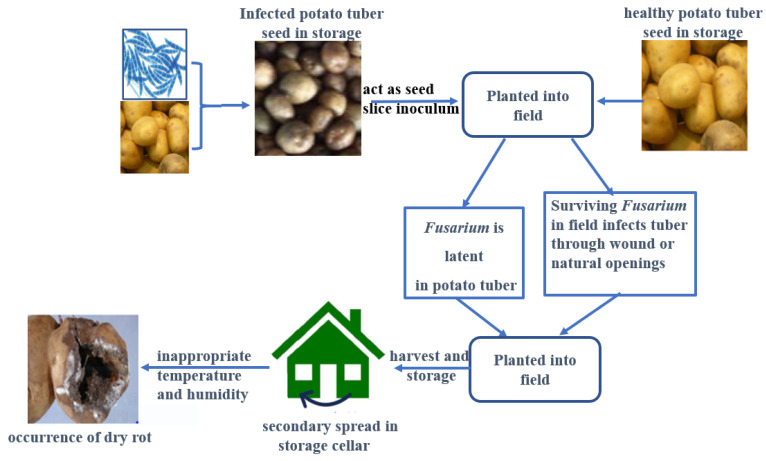
An illustration of the process of *Fusarium* species infecting potato tubers.

In general, *Fusarium* spp. can infect potato tubers through surface wounds or natural openings on tubers during pre-harvest or post-harvest, and the process of *Fusarium* species infecting potato tubers is shown in Figure 1. Pathogenicity is a crucial factor when understanding the pathogens of the *Fusarium* infection of potato tubers. Additionally, extracellular enzymes and reactive oxygen species (ROS) play more important roles for the pathogenicity of *Fusarium*. Pathogens can secrete extracellular enzymes to destroy the cell wall and middle lamellar of the host plant, which makes the pathogen able to spread to the surrounding cell and successfully infect the host plant. Numerous studies indicated that cell wall-degrading enzymes (CWDEs) are important pathogenic factors for *Fusarium* when in infection and spread [29,30]. Yang et al. [29] suggested that the activities of CWDEs (such as polygalacturonase (PG), carboxymethyl cellulose (Cx), polymethyl-galacturonase (PMG), and so on) were found to increase during the *F. sulphureum* infection of potato tubers. Cutinase enzymes were also involved in pathogenicity when *F. solani* infected potato tubers. Moreover, ROS also play a vital role in the pathogenicity of the fungus. As we know, ROS production is an early evens during host-pathogen interactions, and excessive ROS can attack cellular biomolecules, such as lipids, proteins, and DNA, causing cell membrane damage to hosts via lipid peroxidation, and finally leading to infection. For instance, Bao et al. [31] compared the difference in pathogenicity between *F. sambucinum* and *F. sulphureum* during pathogens’ infection of potato tubers, and found that *F. sulphureum* showed higher pathogenicity in inoculated tubers in association with a higher ROS level, which caused a higher malondialdehyde (MDA) content, and a lower level for cell membrane integrity, ultimately leading to a bigger lesion diameter in the inoculated tubers. A contrasting result was observed in tubers inoculated with *F. sambucinum* due to the lower ROS accumulation. Xue et al. [28] also observed that, compared to *F. sambucinum* and *F. solani*, *F. sulphureum* manifested the strongest infection ability and pathogenicity in inoculated tubers (cv. Longshu No.3) in Gansu province. In addition, the pathogenicity of *Fusarium* was also related to host-nonspecific phytotoxin trichothecenes; for instance, fusaric acid produced by *Fusarium* destroyed the cell membrane structure of the host plant, then decreased the respiration rate, which is beneficial for the *Fusarium* infection of potato tubers.

The typical symptoms on the skin of the potato tubers infected by *Fusarium* spp. mainly include a wrinkled brown appearance, and sunken tissue with a dry and leathery appearance. The initial symptoms are observed to be shallow, small brown spots at tuber wound sites after approximately 30 days of storage. Subsequently, the infected tissue begins to become enlarged in every direction, and the tuber’s periderm gradually undergoes subsidence and collapses. Ultimately, concentric rings are observed on the enlarged lesions, and the dead tissue begins to be desiccated [1,32]. A cottony white, purple, yellow, pink, or brick colour of the spores and mycelia of *Fusarium* spp. are observed in the cavity under the rotted lesion [33]. As the disease progresses, whole tubers with symptoms of severe decay always have a shrivelled and dehydrated appearance. In severe cases, the affected potatoes may completely decay, resulting in a mushy texture and foul odour. Therefore, it is important to identify and manage potato dry rot to prevent its further spread and minimize economic losses.

### 2.3. Mycotoxin Accumulation Associated with Fusarium Dry Rot

Potato dry rot resulting from *Fusarium* is associated with mycotoxin accumulation. Mycotoxins are a type of secondary metabolite produced by toxigenic fungi under suitable temperature and humidity conditions, which can lead to a potential health threat to humans and animals [34,35]. The mycotoxin metabolised by *Fusarium* is classified into two types of non-trichothecenes and trichothecenes. The main non-trichothecenes metabolised by *Fusarium* spp. are shown in Table 2. Beauvericin (BEA) and enniatins (ENN) are cyclic hexadepsipeptides with antimicrobial, insecticidal, phytotoxic, and cytotoxic properties, which were detected in potato tubers infected with *F. oxysporum* [36]. Zearalenone (ZEA) and fusarin C (FUS) were detected in tubers infected with *F. sambucinum*, *F. solani*, and *F. oxysporum*, with oestrogenic syndromes in swine and other experimental animals [37,38,39,40]. Fumonisin (FUM) is linked to leukoencephalomalacia in brain lesions of horses and rabbits with hepatotoxic and carcinogenic influences, and is also associated with esophageal carcinoma in humans with phytotoxic effects, which was detected in potatoes contaminated with *F. equiseti*, *F. sambucinum*, and *F. oxysporum* [41]. El-Hassan and Kim [42,43,44] observed sambutoxin (SAM) in potato dry rot infected by *F. sambucinum, F. semitectum*, *F. solani* and *F. oxysporum*, which resulted in haemorrhage in the stomach and intestines, bodyweight loss, apastia, and death for rats. Sonja et al. [45] detected fusaric acid (FA) production in *F. oxysporum*-infected potato tubers, and Venter’s group [46] and El-Hassan [41] indicated that FA content was positively correlated with the incidence of dry rot. Pre- and post-harvest strategies were carried out to control FA accumulation during the dry rot of potato tubers [1].

Trichothecenes are categorised as another main type of mycotoxin found in the Fusarium dry rot of potato that is a type of structurally related sesquiterpene compound. Up to the present, there are more than 190 known trichothecenes.

**Figure 2:**
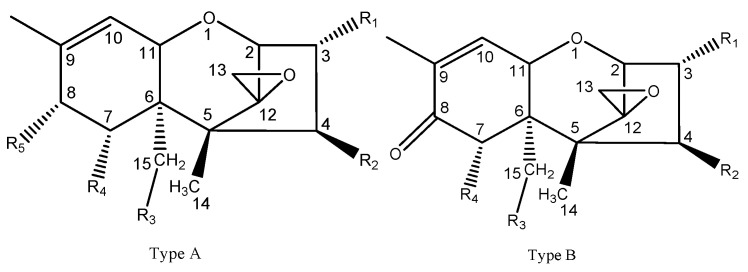

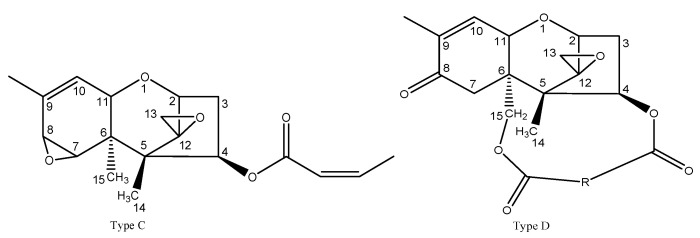
Basic chemical structure of trichothecenes.

Trichothecenes are classified into four different types, A, B, C, and D, based on chemical structural differences; the chemical structures are listed in Figure 2. 

Types A and B of trichothecenes are found in cereal crops and their contaminated products. Additionally, trichothecenes were found in potato dry rot [35], Fusarium dry rot in muskmelon [47], and core rot in apple [48]. As we know, Trichothecenes can pose a serious health threat to humans and animals due to phytotoxicity and mycotoxicoses [28]. For instance, in some severe cases, trichothecenes have potential carcinogenic, teratogenic, and mutagenic effects [49]. Trichothecenes were reported in the Fusarium dry rot of potato tubers (Table 3). Type A and B of trichothecenes are often mainly found in the lesion tissue of rotted potato tubers. Xue et al. [4] found the trichothecenes of 3-ADON, T-2, FUS, and DAS not only in the lesioned part but in the adjacent asymptomatic part of a potato with dry rot contaminated by *F. sulphureum*, *F. solani*, and *F. sambucinum*; it was interesting that the concentration of trichothecenes was negatively correlated with the distance from the infected point. Similarly, Ellner et al. [50] suggested that DAS was detected in rotten tissue, as well as in adjacent asymptomatic tissue in tubers contaminated by *F. sambucinum*, and a similar changing trend to that of Xue’s report was observed. Delgado et al. [51] suggested that DON, NIV, FX, 3-ADON, and 15-ADON were detected in potatoes inoculated with *F. graminearum*, and a similar trend to that in Xue’s and Ellner’s reports was found.

### 2.4. Dry Rot Control

Given the severity of potato dry rot, how to control the disease of dry rot has become an urgent question. Currently, using chemical synthetic fungicides such as thiabendazole, benzimidazole, 2-aminobutane, imazalil, flusilazole, and difenoconazole is the main strategy to control the disease. However, as we all know, a series of problems such as resistance against fungicides, environmental contamination, and pesticide residues have come up, which obliges scientists to develop integrated disease management strategies to combat the problems. An integrated disease control strategy includes potato cultivar selection, and appropriate cultural practices, harvesting processes and post-harvest treatments, and storage conditions along with the application of fungicide pre-harvest or post-harvest.

#### 2.4.1. Variety Screening

The screening of resistant varieties plays a crucial role in controlling post-harvest disease. More than 5000 potato varieties were reported to be planted all over the world [61,62]. Most of the varieties are sensitive to *Fusarium*. Du’s research group investigated 21 potato varieties and 46 breeding lines against *F. sambucinum*, and found that 67 kinds of potato clones were sensitive to *Fusarium* in China [27]. Xue’ group [28] investigated the varieties of Longshu No.6 and Longshu No.3 against *F. sulphureum* and found that the variety of Longshu No.3 showed greater susceptibility to *F. sulphureum*, had more serious disease and higher levels of FUS, DAS, 3ADON and T-2 toxin in potato tubers contaminated by *F. sulphureum* compared to the variety of Longshu No. 6. In Tunisia, Trabelsi et al. [63] indicated that the varieties of Mondial, Spunta, and Liseta were less sensitive to *F. sambucinum*, *F. oxysporum*, and *F. graminearum*. In Iran, Esfahani et al. [64] screened 43 potato varieties to *F. solani*, *F. sulphureum*, and *F. oxysporum*, and found that only the variety of Saturna was resistant against the three fungi. In Canada, Yilma et al. [65] indicated that the variety of Owyhee Russet showed significantly higher resistance than that of Russet Burbank did to dry rot. In fact, resistance to every *Fusarium* spp. is mainly independent and genetically distinct to some extent. The resistance to a species of *Fusarium* is transmitted to progeny but appears to be associated with recessive alleles [66,67]. Even though numerous potato cultivars and clones were tested for sensitivity, no variety is resistant to all the Fusarium species. At the same time, varieties may be sensitive to one species of *Fusarium*; nevertheless, the resistance is to another species of *Fusarium*. Similarly, a certain strain of *Fusarium* maybe pathogenic to one cultivar, but non-pathogenicity is shown for another cultivar because the susceptibility–resistance outcome varies depending on the strains, the varieties, and the prevailing culture and environmental conditions in different regions of the world. Some studies pointed out the role of storage temperature for a cultivar’s susceptibility against *Fusarium* species. Mejdoub-Trabelsi et al. [68] found that cultivars at a temperature of 30 °C were less susceptible, while cultivars at a temperature of 15 °C were highly susceptible. Therefore, it is indispensable to study the populations of *Fusarium* in the field and their pathogenicity to optimize the growth of varieties in each field. At present, breeding resistant cultivars against dry rot is very efficient because of the laborious phenotyping involved [69]. The genome-editing technique using the Clustered Regularly Interspaced Short Palindromic Repeats (CRISPR)/Cas9 system to target genome modifications has become a potential and powerful tool for genetic engineering in potato [70]. The CRISPR/Cas9 system can provide an alternative strategy to that of conventional genetic engineering [71], and is expected to produce disease-resistant cultivars by designing and constructing gene-specific single-guide RNA (sgRNA) vectors.

#### 2.4.2. Cultivation Patterns

Good cultivation patterns play a crucial role and influence the incidence and severity of storage disease after harvest. Generally, cultivation patterns include crop rotation, cultural methods, fertilization, and irrigation. Crop rotation is usually an advised cultural practice in controlling soil-borne disease; however, crop rotation is not very suitable in the management of potato dry rot [72,73]. Because the fungus has a broad host range and can survive in the soil for 5–6 years, it is very difficult to control infection via crop rotation. Potato crop rotation with barley and red clover did not achieve a significant effect for disease incidence and severity in 2–3 years [74].

Cultural methods play a key role in the management of potato dry rot. Dry rot is not only a soil disease but also a tuber-borne disease. Therefore, the seed tuber is usually considered the main source of inoculum [75]. Planting a healthy seed tuber in the field is necessary; planting an infected seed tuber will result in soil infestation around the progeny of the tuber [76]. The contaminated soil adhering to the tuber’s surface will eventually contaminate tubers through wounds or natural openings during storage after harvest (Figure 1). A reasonable sowing time is also an important factor for potatoes, and allows the whole growth period of a potato to have a relatively suitable temperature and humidity, avoiding high temperatures when a potato tuber develops and expands. Finally, reasonable fertilization and irrigation should be considered during different growth periods.

#### 2.4.3. Harvesting, Grading and Packing

Harvesting and processing treatment after harvest significantly influence the control effect disease. Because the fungus of *Fusarium* spp. attacks potato tubers mainly through wounds, considerable efforts should be focused on avoiding tuber bruising and injuring when harvesting [15]. In addition, a temperature of 10–18 °C for tuber pulp is the best option for harvesting tubers [77]. The maturity level plays an important role when harvesting. Tubers with low maturity have a higher content of sucrose and a poorer skin set; however, a higher level of sucrose provides nutrition for fungus growth, and poorer skin is prone to bruising and the generation wounds. These properties lead to potatoes with low maturity that are more vulnerable to the fungus [18]. In general, it is appropriate for potato tubers to be harvested after 7–14 days of killing the potato vine, which is a sufficient amount of time for wound healing and decreases the chances of fungus attack [15]. It takes 1–2 weeks for A tuber to heal the wound when the environmental humidity is between 95 and 99%, and the tuber pulp temperature ranges from 13 to 16 °C, which is favours the rapid healing of a wound after harvest. Taking some steps to accelerate wound healing not only saves the wound healing time but also decreases labour costs. Our research group’s previous results suggested that sodium silicate or brassinosteroid treatment accelerated the wound healing process of tubers via the activation of phenylpropanoid metabolism [78,79]. Subsequently, Jiang adopted transcriptomics analysis of benzo-(1, 2, 3)-thiadiazole-7-carbothioic acid s-methyl ester (BTH) to induce genes involved in suberin accumulation to accelerate the potato wound healing process [80]. Moreover, a careful examination of tuber grading, and packing should be paid attention to. As mentioned above, the disease of dry rot can easily contaminate potatoes through wounds; when one tuber decays, the rotten tuber will contaminate the tubers around it, which will ultimately result in a disastrous disease during storage. Therefore, tubers with wounds (including pests and disease appearance) must undergo a thorough examination and rejected before storage. Actually, the examination process requires proper grading before storage [81]. Finally, packing can not only reduce disease incidence, but can also increase the beauty and value of potatoes.

#### 2.4.4. Storage Cellar Management

Storage cellar management is a crucial factor in managing the dry rot of potato, and includes storage cellar disinfection, and temperature, humidity, and gas components. During storage, proper disinfection treatment for storage facilities is mandatory, and the common chemical disinfectants include sulphur, potassium permanganate-formaldehyde, peracetic acid, chlorine dioxide, and 2–4% formalin solution. Most of these chemical disinfectants are employed for fumigation processing. Storage temperature is the determining factor of the storage quality of potato tubers. The storage temperature should be decreased to an appropriate level after wound healing at 15–20 °C. In general, the storage temperature is 2–3 °C for seed potatoes and 4–5 °C for commercial potatoes. During storage, the appropriate humidity is 80–93%, and higher humidity will lead to tuber rot and sprouting earlier. In addition, proper cool air circulation is also essential, because stored potato tubers produce excessive carbon dioxide (CO_2_) and heat, which will facilitate the adherence of *Fusarium* to spores [82].

#### 2.4.5. Physical, Chemical, and Biological Treatment

##### Physical Treatment

As we know, with minimal environmental impacts and no residues in the treated product, the development of a physical application for the management of post-harvest plant diseases has been widely carried out. Among all kinds of physical treatments, the extensive application of ultraviolet-C light (UV-C, 190–280 nm) showed a significant effect; on the one hand, UV-C treatment can directly suppress pathogen growth, and on the other hand, UV-C application can induce a defence response in host tissues and increase resistance against disease [83]. The application of UV-C has been shown to control dry rot by inducing the generation of antifungal substances in potato that contribute to disease control [84]. Ranganna et al. [85] also suggested that UV-C irradiation completely prevents the development of dry rot caused by *F. solani* in potatoes under storage at 8 °C for 3 months. Yu et al. [86] indicated that 35 kJ·m^−2^ UV-C treatment significantly inhibited the dry rot of potato by increasing the activities of CAT, POD, and PAL. Another report was written by Jakubowski and Krolczyk [87], who suggested that UV-C radiation effectively controlled the disease of dry rot in stored potato tubers by inhibiting the development of fungi.

##### Chemical Treatment

The most effective strategy to control potato dry rot is the combination of pre- and post-harvest treatment. Firstly, disinfection the seed tuber before planting, and application of chemical treatments after planting plays an important role for management the dry rot. Thiabendazole is currently considered the most widely used chemical fungicide to manage the Fusarium dry rot of potato [88]. Thiophanate-methyl (benzimidazole group) was reported to be extensively applied to manage seed tuber piece disease in Canada. Nevertheless, the employment of thiabendazole has resulted in the occurrence of the drug-resistant strains against the pathogen of *F. sambucinum*, and it is fortunate that the rest of the *Fusarium* species viz. *F. solani*, *F. culmorum*, *F. equiseti*, *F. acuminatum* and *F. avenaceum* are still sensitive to the fungicide of thiabendazole [72,89]. Some alternative fungicides with high efficiency and low toxicity (such as fludioxonil) also have incomparable effects in managing dry rot. For instance, fludioxonil was used to effectively control tuber seed disease and sprout rot [90]. The application of azoxystrobin and fludioxonil effectively managed dry rot, and the disease incidence decreased to 50% comparing to that of the control after 21 days of storage [15]. However, with the extensive application of synthesised chemical fungicides, the inevitable problems of drug resistance and environmental pollution, as well as food safety are becoming more and more prominent. Therefore, it is an urgent need to develop more safe and efficient fungicides to control the Fusarium dry rot of potato.

Accordingly, generally-recognised-as-safe (GRAS) substances, such as inorganic acids, organic acids, inorganic salts, organic salts, essential oils and phytohormones, all display excellent effects in terms of sustainably controlling the dry rot of potato. Raigond et al. [91] indicated that chitosan application significantly managed dry rot in potato, and he also found that a chitosan coating significantly reduced *Fusarium* incidence by inhibiting *Fusarium* growth. Xue et al. [7] suggested that chitosan, sodium silicate and β-aminobutyric acid treatments markedly inhibited the expansion of lesion diameter in tubers infected with *F. sulphureum*. Interestingly, the trichothecene concentration was also decreased; the involved action mechanism was attributed to the up-regulation of enzyme activities involved in the defence response, and down-regulated genes were related to the trichothecene biosynthesis pathway. Afterwards, Xue’s research group found an interesting result that T-2 toxin, as a kind of trichothecene A, suppressed the spread of dry rot of potato at a low concentration [92]. Later, Han found that the treatments of sodium silicate and brassinosteroid respectively promoted the wound healing of potato and accelerated suberin deposition, ultimately enhancing resistance against the fungi [78,79]. Jiang adopted BTH to treat potato and also observed a similar control effect on potato dry rot, with accelerated wound healing [80]. Ma et al. [93] found that the gene *StCDPK23* played an important role in the wound healing of potato and suberin deposition, and constructed *StCDPK23*-overexpressing plants to conform to *StCDPK23* to participate in tuber wound healing and contribute to resistance against the dry rot of potato.

In addition, essential oils and extracts from plants display an excellent effect in that they suppress the development of Fusarium dry rot via soaking or fumigation treatment [94]. The essential oil of *Zanthoxylum bungeanum* was found to be efficient in inhibiting the expansion of dry rot disease resulting from *F. sulphureum* [11]. Cinnamaldehyde, a major component of cinnamon essential oil, displayed a better control effect on potato dry rot resulting from *F. sambucinum*; the underlying mechanism revealed that cinnamaldehyde suppressed spore germination by impacting the biosynthetic pathway of ergosterol, improving ROS accumulation, and ultimately resulting in a breakdown of cell membrane integrity [12]. Similarly, the essential oils from peppermint and fennel also remarkedly suppressed the growth of *F. oxysporum in vitro*, and inhibited the development of potato dry rot when treated with a protective emulsifiable concentrate [95]. It is interesting that some essential oils directly influenced mycotoxin metabolism by impacting the biosynthetic pathway of mycotoxins; for instance, the essential oils from palmarose and clove reduced DON and ZEA accumulation by down-regulating the expression of genes involved in mycotoxicity in the biosynthetic pathway of DON and ZEA [96]. Essential oils from plants, as a sustainable alternative to chemical synthetic fungicides, need to be studied in-depth in the future [1,97]. Plant extracts also display excellent effects on plant disease [98]. The extract from black spruce revealed antifungal and suppressive potential to prevent the development of potato dry rot [99]. The extract from cinnamon also significantly inhibited *F. sambucinum* spore growth in vitro and reduced dry rot development in vivo [100]. Chlorogenic acid, as a kind of polyphenol with antioxidative activity, is mainly sourced from methanol extract, and also displayed better inhibitory activity on the development of Fusarium dry rot of potato. The possible action mechanism is attributed to the alteration of the morphological structure of *F. sambucinum* after chlorogenic acid application, and the changing of curling, twisting and collapse were observed after exposure to chlorogenic acid [1].

Additionally, chlorine dioxide and ozone, as the two strong oxidants, also perform important and crucial roles in controlling Fusarium dry rot. Chlorine dioxide (ClO_2_) acts as a water-soluble strong oxidant, whose oxidation ability is 2.5 times higher than that of chlorine. ClO_2_ can be applied in both a gaseous form and as an aqueous solution to control post-harvest disease in fruits and vegetables. For instance, Li et al. [101] suggested that the application of 0.75 ug/mL of ClO_2_ solution significantly reduced the incidence of Fusarium dry rot of potato and suppressed the expansion of the lesion diameter by damaging the morphology and ultrastructure of *F. sulphureun* hyphae. Ozone, as another high-efficiency and non-toxic, strong oxidant, also display an important role in controlling potato dry rot; on the one hand, ozone inhibited the growth of *F. sulphureum* spores, and destroyed the structure of *F. sulphureum* [102], while on the other hand, ozone treatment activated the ROS metabolism of potato tubers, and induced resistance against dry rot [103].

**Figure 3:**
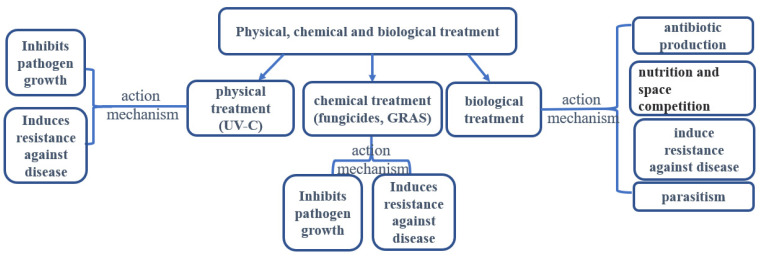
The strategy of controlling potato dry rot via physical, chemical and biological methods and the possible action mechanism.

In fact, the possible action mechanism of chemical treatment was attributed to two facts: on the one hand, chemical treatments inhibit the growth of pathogens; on the other hand, chemical treatments induce resistance against the dry rot of potato tubers (Figure 3).

##### Biological Treatment

Biocontrol is regarded as a greener and safer strategy for food safety and human health, comparing to traditional chemical synthetic fungicides. Presently, scientists have been focusing on research on antagonistic microorganisms to manage plant diseases. Antagonistic microorganisms currently regarded as the best potential alternative option with which to manage post-harvest diseases. For instance, antagonistic microorganisms effectively controlled Fusarium dry rot during the potato wound-healing process when tubers were at their most vulnerable. Schisler’s group firstly reported the strains of *Pseudomonas Migula* spp., *Enterobacter Hormaeche* and *Edwards* spp., and *Pantoea Gavini*. spp. remarkedly decreased the incidence of potato dry rot resulting from *F. sambucinum* [104]. Later, the group found that mixtures of various antagonist strains were more efficient in controlling potato dry rot than a single strain [105]. Gözdenur and Elif [106] screened 12 bacterial and fungal isolates and found that *Pantoea agglomerans* had the best efficacy in suppressing the growth of *F. oxysporum* and in controlling the occurrence of potato dry rot.

*Trichoderma harzianum* and *Bacillus subtilis*, as the two most important biological control agents, were registered to manage potato disease. The two agents were the most studied mycoparasitic species for their antagonistic function against a broad spectrum of pathogenic fungi, as well as being recognised as the most promising strategies to inhibit different kinds of pathogenic fungus growth and to control both pre-harvest and post-harvest plant diseases occurrence. Daami-Remadi et al. [107] suggested that *T. harzianum* and *T. viride* manifested greater antagonistic activity against Fusarium dry rot in potato in Tunisia. El-Kot [108] compared four strains of fungal, bacterial and bioagent actinomycetes, and suggested that *T. harzianum* displayed the best efficiency in inhibiting the radial development of *F. sambucinum* and controlling the occurrence of dry rot in a greenhouse. Paul et al. [109] also observed that the *T. harzianum* strains CMML20-26 and CMML20-27 significantly decreased post-harvest disease in sweet potato. *B. subtilis* also plays a vital role in reducing potato disease. Wharton and Kirk [110] used the bioagent of *B. subtilis* in combination with excellent management practices to significantly reduce seed piece decay by 94.3% in 2007. Hussain et al. [111] compared the biosurfactant extract, culture filtrate and bacterial cell suspension from *B. subtilis HussainT-AMU* and found that the bacterial cell suspension (49%) and biosurfactant extract (70%) had the most control effects on the net house and field, respectively.

In recent years, *Trichothecium roseum* was also reported to act as an elicitor to induce resistance against dry rot in potato tubers infected by *F. sulphureum*. During the defence responses induced by the elicitor, the genes involved in a resistant reaction were up-regulated; accordingly, the enzymes activities and antifungal compound contents were also significantly decreased after *T. roseum* application [112]. The possible mode of action for the bioagent includes mycoparasitism, competition for nutrients and spaces, and the production of extracellular enzymes and/or secondary metabolites (such as antibiotic compounds and mycotoxins) (Figure 3) [113]. For instance, Xue’s research group [92] indicated that T-2 toxin (secondary metabolites from *F. sulphureum*) at the concentration of 1µg/mL could act as an elicitor to induce resistance against dry rot by activating ROS metabolism and phenylpropane metabolism in potato.

## 3. Conclusions

Fusarium dry rot of potato is regarded as a major form of disastrous potato decay that damages tuber quality and causes economic losses and mycotoxin contamination. There are 17 species and 5 variants of *Fusarium* causing potato dry rot all over the world, and the changes in genetic diversity depend on geographical locations. The frequency of occurrence and aggressiveness of Fusarium dry rot also differ depending on the prevalent variety and ambient conditions in a location-specific manner. Because the susceptibility or resistance of a particular cultivar is related to *Fusarium* species and storage temperature, a breeding project urgently needs to be designed, for different cultivars to adapt against *Fusarium* species.

To efficiently control the occurrence of the Fusarium dry rot of potato, an integrated disease-controlling strategy is recommended that includes excellent harvesting conditions to avoid tuber injury and suitable storage conditions (optimum temperature, humidity, and CO_2_ concentration), as well as planting seed tubers free of visible disease, and registered chemical synthetic fungicide and/or post-harvest GRAS treatment. Fusarium dry rot-controlling strategies eventually integrate the application of alternatives such as GRAS and microbial antagonists. Efficient strategies to manage Fusarium dry rot mainly depend on further research such as a combination of gene editing and molecular breeding, as well as development efforts between scientists and industry to implement an integrated management measure towards the high-efficiency control of the Fusarium dry rot of potato.

## Figures and Tables

**Table 1:** The reported *Fusarium* spp. to lead to dry rot of potato in diverse countries and regions.

*Fusarium* Species	Region	Reference
*F. sambucinum*	North American and some regions of Europe	[16,17,18]
*F. coeruleum*	United Kingdom and Great Britain	[19,20,21]
*F. graminearum*	North Dakota	[22]
*F. solani* and	South Africa	[23,24]
*F. oxysporum*	Michigan	
*F. sulphureum* and *F. solani*	Iran	[25]
*F. sambucinum*	Egypt	[26]
*F. oxysporum*		
*F. verticillioides*		
*F. incarnatum*		
*F. avenaceum*,	Heilongjiang Province and Inner Mongolia Autonomous Region	[27]
*F. oxysporum*,		
*F. sporotrichiodes*		
*F. solani*,		
*F. trichothecioides*,		
*F. solani* var. *coeruleum*		
*F. sambucinum*		
*F. semitectum*,		
*F. solani*		
*F. sambucinum*		
*F. culmorum*,		
*F. gibbosum*,		
*F. macroceras*,		
*F. solani* var. *coeruleum*, *F. acuminatum*,		
*F. equiseti* and *F. redolens*		
*F. sambucinem*	North of China	[27]
*F. avenaceum*	Shanxi Province	
*F. solani* var. *coeruleum*,		
*F. oxysporum*,		
*F. acuminatum*		
*F. sambucinem*	Northwest of China,	[7,8,10,11,12,27]
*F. avenaceum*	Gansu Province, Ningxia Hui Autonomous Region	
*F. graminearum*		
*F. solani*		
*F. sulphureum*		
*F. tricinctum*,	Northwest of China, Qinghai Province	[6]
*F. avenaceum*,		
*F. oxysporum*,		
*F. solani*,		
*F. acuminatum*,		
*F. equiseti*		
*F. solani*,	South of China Zhejiang Province	[6]
*F. moniliform*,		
*F. redolens*		

**Table 2:** Non-trichothecenes generated by *Fusarium* spp. in potato dry rot.

*Fusarium* Species	Non-Trichothecenes	Reference
*F. oxysporum*	BEA, ENNs	[36]
*F. sambucinum*	ZEA, FUS	[37]
*F. solani*	ZEA, FUS	[37]
*F. oxysporum*	ZEA, FUS	[37]
*F. crookwellense*	ZEA, FUS	[38]
*F. crookwellense*	ZEA, FUS	[39]
*F. crookwellense*	ZEA, FUS	[40]
*F. graminearum*	ZEA	[40]
*F. equiseti*	FUM, ZEA	[41]
*F. oxysporum*	FA, FUM, ZEA	[41]
*F. sambucinum*	SAM	[42]
*F. sambucinum*	SAM	[43]
*F. sambucinum*	SAM	[44]
*F. oxysporum*	FA	[45]
*F. oxysporum*	FA	[46]

Note: BEA: beauvericin, ENNs: enniatins, ZEA: zearalenone, FA: fusaric acid, FUM: fumonisin, FUS: fusarin C, SAM: sambutoxin.

**Table 3:** Trichothecenes generated by *Fusarium* spp. in potato dry rot.

*Fusarium* Species	Trichothecenes	Reference
*F. sambucinum*	trichothecene	[37]
*F. solani*	trichothecene	[37]
*F. oxysporum*	trichothecene	[37]
*F. crookwellense*	NIV, FX	[38]
*F. sambucinum*	DAS	[39]
*F. crookwellense*	NIV, FX	[39]
*F. sulphureum*	3-ADON, T-2, FUS, DAS	[4]
*F. solani*	3-ADON, T-2, FUS, DAS	[4]
*F. sambucinum*	3-ADON, T-2, FUS, DAS	[4]
*F. equiseti*	T-2	[41]
*F. oxysporum*	T-2	[41]
*F. sambucinum*	DAS	[50]
*F. sambucinum*	DAS, MAS, NEO, T-2, HT-2	[52]
*F. sambucinum*	4,15-DAS, 15-MAS, 4-MASc	[53]
*F. solani*	DON, HT-2, 3-ADON	[54]
*F. sambucinum*	DON, NIV, HT-2	[54]
*F. sambucinum*	MAS, DAS	[55]
*F. crookwellense*	NIV, DAS	[56]
*F. culmorum*	NIV, FX, 3-ADON, DON	[57]
*F. crookwellense*	FX	[57]
*F. equiseti*	NIV, FX, 4-MAS, 15-MAS, DAS, SCR	[57]
*F. graminearum*	NIV, FX, DON, 3-ADON, 15-ADON	[57]
*F. sambucinum*	SCR, 4-MAS, 15-MAS, DAS, NEO, HT-2,T-2	[57]
*F. graminearum*	DON, NIV, FX, 3-ADON, 15- ADON	[51]
*F. graminearum*	NIV, T-2, 3-ADON, 15-ADON,15-SCR	[58]
*F. graminearum*	DON, 3ADON, 15-ADON	[59]
*F. culmorum*	DON, 3-ADON	[60]

Note: DAS: diacetoxyscirpenol; 4,15-DAS: 4,15-diacetoxyscirpenol; DON: deoxynivalenol; 3-ADON: 3-acetyldeoxynivalenol; 15-ADON: 15-acetyldeoxynivalenol; HT-2: HT-2 toxin; MAS: mono-acetoxyscirpenol; 4-MAS: 4-acetyl-monoacetoxyscirpenol; NIV: nivalenol; FX: fusarenone X; NEO: neosolaniol; SCR: scirpentriol; 15-MAS: 15-acetyl-monoacetoxyscirpenol; T-2: T-2 toxin; 15-SCRP: 15-acetylscripenol.

## Data Availability

Not applicable.
